# Tea-Leaves as an Application to Burns and Scalds

**Published:** 1871-04

**Authors:** W. H. Searles

**Affiliations:** Warsaw, Wis.


### TEA-LEAVES AS AN APPLICATION TO BURNS AND SCALDS.

BY W. H. SEARLES, M.D., Warsaw, Wis.

Some few years since, I accidentally found that a poultice of tea-leaves, applied to small burns and scalds, afforded immediate relief, and I determined to give it a more extensive trial when opportunity should present, and which soon occurred.

It was in a case of a child 14 months old. Upon examination, I found the entire anterior portion of the body, arms, and legs blistered and deeply burned from a kettle of hot water which the child had upset upon itself. The case, to say the least, was unfavorable for the success of any remedy.

I prepared a large poultice, softening the leaves with hot water, and, while yet *quite warm*, applied it upon cotton-wool, over the entire burned surface.

Almost like magic, the sufferings abated, and, without the use of *any other anodyne*, the child soon fell into a quiet sleep.

In a few hours I removed the application, and re-applied where it was necessary. I found the parts discolored and apparently *tanned.* The acute sensibility and tenderness had nearly disappeared, and the little patient passed through the second and third stages under far more favcrable circumstances (symptoms) than was at first anticipated, making a recovery in about two weeks. Since then, on several occasions, I have had reason to commend tea-leaves, till now I have come to prefer it above all other remedies in the first stage of burns and scalds.

I think it must recommend itself to the profession, not only on account of its intrinsic worth, but also by reason of its great convenience, being so readily obtained.

I am not aware that any mention has been made thus far of this article in this connection, and I hope that others will find it as useful in their hands as the writer has.

